# Normal sperm quality despite partial deletion of sY84 in the AZFa region: A case report

**DOI:** 10.1097/MD.0000000000039481

**Published:** 2024-08-30

**Authors:** Derong Li, Bowen Luo, Yudi Luo, Keng Feng, Xiang Li, Lingling Zhu, Luhai Ruan, Fuman Cai, Yujuan Liao, Ling Li

**Affiliations:** a Reproductive Medicine Center, Yulin Maternal and Child Health Care Hospital, Yulin, Guangxi, China.

**Keywords:** AZFa, sY84, Y chromosome microdeletion

## Abstract

**Background:**

The complete absence of the azoospermia factor A (AZFa) region typically results in nonobstructive azoospermia. Partial deletions of the AZFa region are particularly noteworthy due to the limited and enigmatic reports of partial deletions in the AZFa region. Here, we present a rare case report of partial deletion of sY84 in the AZFa region but exhibiting normal sperm quality. The aim of this case report is to gain a deeper insight into the impact of AZFa region deletion on male fertility and to guide future clinical decisions and treatment strategies.

**Methods and Results:**

A 25-year-old man presented to the hospital with his 25-year-old wife due to recurrent spontaneous abortions. Routine semen analysis, sperm morphology analysis, acrosomal enzyme analysis, sperm DNA fragmentation indexed, and peripheral blood karyotype analysis revealed no abnormalities. Y chromosome microdeletion was detected by real-time fluorescence polymerase chain reaction, which showed that sY84 could not be amplified and sY86 was amplified nonspecifically. The man was diagnosed with partial deletions in the AZFa region. The wife underwent in vitro fertilization treatment for tubal infertility and recurrent spontaneous abortions. The couple successfully delivered a healthy daughter weighing 2.7 kg at 39 weeks of gestation, following 2 assisted reproductive pregnancies.

**Conclusion:**

Our findings contribute to expanding our knowledge of the AZFa region. A sY84 deficiency in the AZFa region may not lead to spermatogenesis failure and may potentially be one of the factors causing recurrent miscarriages, which needs to be confirmed by further data.

## 1. Introduction

Infertility is a global issue affecting around 15% to 20% of couples.^[1]^ Male factor infertility accounts for approximately half of the cases.^[2,3]^ Genetic causes, such as Klinefelter syndrome, translocations, deletions, and Y chromosome (Yq) microdeletions, have been identified as primary factors contributing to male infertility.^[4,5]^ Yq microdeletions, which refer to deletions within the male-specific region of the Yq, primarily occur in the long arm of the Yq within a specific region called azoospermia factor (AZF). The AZF region consists of 3 subregions, namely AZFa, AZFb, and AZFc.^[6]^ Yq microdeletions may result in both spermatogenesis failure and recurrent pregnancy loss (RPL).^[7]^ In addition, Yq microdeletions can always be inherited from the father to the son. The prevalence of AZF microdeletions in infertile men ranges from 12% to 61.33%,^[8]^ while deletions in the AZFa region represent only 0.1% to 3.8% of the Yq microdeletions.^[9,10]^ The AZFa microdeletions are associated with male infertility and spermatogenic failure.^[11]^ Complete deletions in the AZFa region result in azoospermia and Sertoli cell-only syndrome.^[12]^ However, cases of partial AZFa deletions are rare and poorly understood. Here, we report a case with normal sperm quality despite partial AZFa deletions.

## 2. Case presentation

### 2.1. Patient information

A 25-year-old man and his 25-year-old wife presented to the Reproductive Medicine Center at the Yulin Maternal and Child Health Care Hospital for recurrent spontaneous abortion. There was no family history of infertility. The individual measured 165 cm in height, weighed 56 kg, and had a body mass index of 20.57 kg/m². He had no history of previous illnesses and no apparent intellectual disability. The volume of each testis was 12 mL. No disorders were found in the seminal ducts or epididymis, and there was no evidence of varicocele or gynecomastia.

### 2.2. Diagnostic workup and diagnosis

**Figure 1.**
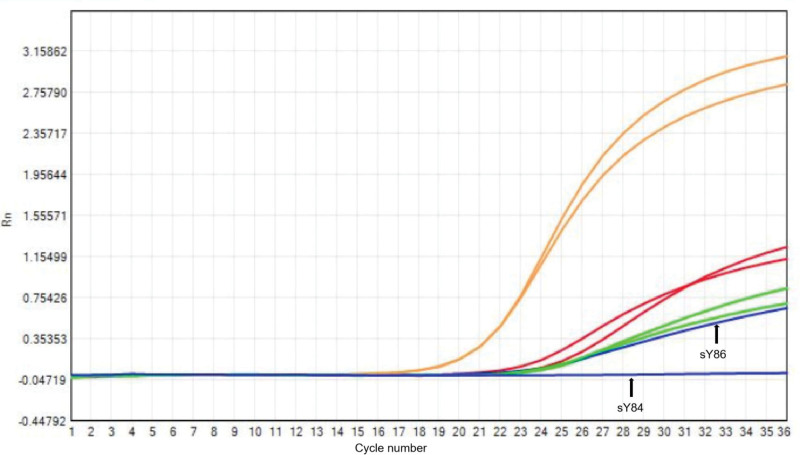
Amplification curves for a partial AZFa microdeletion. sY84, sY127, sY255, sY86, sY134, and sY254 amplification curves. The AZFa marker (sY84) was absent, while another marker (sY86) was detected. AZFa = azoospermia factor A.

The semen analysis results were as follows: sperm concentration was 24.95 × 10^6^/mL, with 72.35 × 10^6^ spermatozoa per ejaculation. Normal morphology was 3.5%, vitality was 59%, progressive motility was 36.36%, total motility was 47.35%, acrosomal enzyme was 206.8 µIU/10^6^, and sperm DNA fragmentation index was 15%. The hormonal findings were as follows: follicle-stimulating hormone at 2.08 mIU/mL (normal value, 0.95–11.95 mIU/mL), testosterone at 4.94 ng/mL (normal value, 1.42–9.23 ng/mL), luteinizing hormone at 1.16 mIU/L (normal value, 0.57–12.07 mIU/mL), prolactin at 18.74 ng/mL (normal value, 3.46–19.4 mIU/mL), and estradiol at 23 pg/mL (normal value, 11–44 pg/mL). Chromosome studies on cultured lymphocytes showed a 46,XY karyotype. Following the guidelines of the European Academy of Andrology/European Molecular Genetics, 6 sites were selected for preliminary screening for AZF deficiency, including SY84/SY86 (AZFa), SY127/SY134 (AZFb), and SY254/SY255 (AZFc) for the identification of Yq microdeletions. The results of this case polymerase chain reaction testing revealed the presence of sY127 and sY134 within the AZFb region, sY254 and sY255 within the AZFc region, and sY86 within the AZFa region. However, sY84 was not identified in the AZFa region (Fig. 1). And deletion of AZFa region sequence-tagged site (STS) sY84 was confirmed by repeated analysis. The patient was diagnosed with Yq microdeletion, a condition closely associated with male infertility and recurrent miscarriage. Given the history of recurrent miscarriage in the couple, our attention was drawn to the possibility of Yq microdeletion being a contributing factor. It is plausible that Yq microdeletion is closely linked to recurrent miscarriage in this particular couple.

### 2.3. Treatment and outcome

The wife underwent in vitro fertilization treatment for tubal infertility and recurrent spontaneous abortions. The wife successfully delivered a healthy daughter weighing 2.7 kg at 39 weeks of gestation, following 2 assisted reproductive pregnancies. The patient provided written informed consent. This study was approved by the Ethics Committee of Yulin Maternal and Child Health Care Hospital.

## 3. Discussion

Microdeletions at the AZF locus on the Yq represent the second most prevalent genetic factor leading to male infertility, following Klinefelter syndrome, which is present in 10% to 15% of infertile men with nonobstructive azoospermia or severe oligospermia.^[13]^ The AZF region comprises 3 distinct subregions: AZFa, AZFb, and AZFc. AZFc deletion is the most prevalent form of complete deletion, making up 70% to 80% of cases, while AZFa accounts for 0.5% to 9%, AZFb for 1% to 7%, and AZFbc for 1% to 20%.^[14]^

The complete deletion of the AZFa region leads to the development of Sertoli cell-only syndrome, characterized by the presence of only Sertoli cells and the absence of sperm.^[12]^ Partial deletion of AZFa is rare. The precise number of genes/loci within the AZFa region that contribute to reduced sperm production remains uncertain at present. Additionally, little is known about the various phenotypic manifestations observed in individuals with partial deletions of AZFa. The European Academy of Andrology and the European Molecular Genetics Quality Network suggest that sY84 and sY86 are the most important STS primers that should be used in the evaluation of AZFa deletion.^[14]^ The deletion indicates a high probability of complete AZFa deletion. The STS marker, sY84, which is located within the AZFa region, is positioned upstream of the DEAD-box helicase 3, Y-linked (*DBY*) gene.^[15]^ The deletion of sY84 in the AZFa region results in the absence of the *DBY* gene. The *DBY* gene is specifically expressed in testicular tissue. The absence of this gene leads to pathological phenotypes in testicular cells, causing growth disorders in germ cells.^[16]^ Additionally, the mean levels of sex hormones in azoospermic sterile patients with AZF microdeletion were found to be higher than the mean levels in azoospermic infertile males without AZF microdeletions. Furthermore, the quality and quantity of sperm in patients with AZF microdeletion decrease with age.^[17]^ Therefore, the investigation of AZF microdeletions in the Yq is significant in the context of male infertility. In our study, we found sY84 deletion in the AZFa region in the reported patient. The patient’s clinical phenotype was normal, including hormonal levels, peripheral blood karyotype, testis size, and semen quality, suggesting that the deletion of sY84 in the AZFa region may not lead to spermatogenesis disorders. Additional research is required in order to establish a more precise definition of this particular patient population, as the availability of comprehensive datasets is currently limited.

RPL affects approximately 2.5% of women and is defined as 2 or more clinical pregnancy losses, encompassing both embryonic and fetal loss prior to gestational weeks 20 to 24.^[18]^ Numerous factors influence RPL. Approximately 50% of couples have no explanation for their RPL. In recent times, there has been increasing attention toward the significance of male factors in causing RPL. Yq microdeletions, epigenetic modifications, paternal age, and anomalies in sperm chromosome structure and number have been previously linked to RPL.^[19,20]^ Several studies suggest that microdeletion of Yq is a cause ofRPL.^[7,20]^ In this study, the couple presented to the Reproductive Medicine Center at the Yulin Maternal and Child Health Care Hospital for recurrent spontaneous abortion. The wife underwent in vitro fertilization treatment for tubal infertility. They had a daughter after 2 assisted reproductive pregnancies. Interestingly, the couple suggests that Yq microdeficiency may be associated with recurrent abortion.

More evidence is needed to support the conclusion, as this is a retrospective case report of a rare disease and the family lineage was not investigated. Research on Yq microdeletions may offer new insights into the study of couples affected by recurrent miscarriages.

## 4. Conclusions

The patient described in this article was found to have a deletion of the sY84 site within the AZFa region. However, the patient’s clinical phenotype appeared normal, suggesting that the deletion of the sY84 site within the AZFa region may not lead to spermatogenesis failure. Furthermore, it is plausible that RPL could be associated with deletions involving the sY84 site within the AZFa region. Given the limitations imposed by small sample sizes, further investigation and substantial clinical data are required to confirm and elucidate any potential relationship between rare microdeletion types in this specific region of Yq and RPL.

This article enriched our understanding of Yq microdeletion, and research on Yq microdeletions may offer new insights into the study of couples affected by recurrent miscarriages in future.

## Acknowledgments

The authors thank all the peer reviewers for their opinions and suggestions.

## Author contributions

**Writing – original draft:** Derong Li.

**Writing – review & editing:** Bowen Luo, Yudi Luo.

**Data curation:** Keng Feng, Xiang Li, Lingling Zhu, Luhai Ruan, Ling Li.

**Conceptualization:** Fuman Cai, Yujuan Liao.
